# Altered gut microbiota and serum metabolite profiles characterize postmenopausal bone loss: insights into the gut-bone axis

**DOI:** 10.3389/fmicb.2026.1750495

**Published:** 2026-03-04

**Authors:** Zhiming Guan, Yanpin Liu, Junying Zhao, Longlong Jia, Yanyan Zhao, Hang Pan, Lijun Chen

**Affiliations:** 1 School of Public Health, Shandong Second Medical University, Weifang, Shandong, China; 2 National Engineering Research Center of Dairy Health for Maternal and Child, Beijing Sanyuan Foods Co. Ltd., Beijing, China; 3 Beijing Engineering Research Center of Dairy, Beijing Technical Innovation Center of Human Milk Research, Beijing Sanyuan Foods Co. Ltd., Beijing, China

**Keywords:** bone mineral density, gut microbiota, gut microbiota-bone link, N-acetylanthranilic acid, postmenopausal bone loss, *Prevotella*, serum metabolomics, vitamin D

## Abstract

**Introduction:**

Postmenopausal bone loss is a multifactorial condition influenced by hormonal changes, metabolic dysregulation, and gut microbiota alterations. Emerging evidence indicates the potential significance of the gut microbiota-bone link in maintaining bone homeostasis. The present study investigated the composition of the gut microbiota and serum metabolite signatures of postmenopausal women afflicted with bone loss, as well as the interrelationships between these factors, to explore the potential associations of the gut microbiota-bone link.

**Method:**

In total, 105 postmenopausal women from Beijing were classified by DXA into a bone loss group (L1–L4 *T*-score <−1.0; *n* = 58) and a normal bone mass group (L1–L4 *T*-score ≥−1.0; *n* = 47). Gut microbiota composition was assessed by 16S rRNA sequencing and serum metabolites by UHPLC-MS/MS. Differential abundance and Spearman correlation analyses were performed in relation to BMD/*T*-scores and serum biochemical indicators.

**Results:**

Compared with controls, the bone-loss cohort showed lower BMD and *T*-scores at L1–L4 and at the femoral neck, and a longer period since menopause. Additionally, the bone-loss cohort exhibited modestly higher, yet still subnormal, circulating 25(OH)D and 25(OH)D3 concentrations, which were inversely associated with L1–L4 *T*-scores. The bone loss group was characterized by a diminished abundance of protective genera *Prevotella* and *Dorea* and increased levels of Limosilactobacillus and Olsenella. Prevotella and Dorea showed positive trends with L1–L4 *T*-scores but did not reach statistical significance. Metabolomic analysis identified 33 differential metabolites, with higher levels of flavonoids (taxifolin), L-arginine, and spermidine in the normal bone group and reduced lysophosphatidylcholine levels. N-acetylanthranilic acid (NAA) was positively correlated with L1–L4 *T*-scores and the relative abundances of Prevotella and Dorea.

**Discussion:**

Postmenopausal bone loss is associated with gut microbiota alterations and altered serum metabolic profiles. Although circulating 25(OH)D levels were relatively higher (yet still subnormal) in the bone-loss group, this cross-sectional observation should be interpreted as an association rather than evidence of a compensatory mechanism. Our findings indicate that NAA and its associated taxa are correlationally associated with bone-related phenotypes, supporting a testable microbiota-metabolite hypothesis that warrants validation in longitudinal or interventional studies.

## 1 Introduction

Bone remodeling is a continuous physiologic process that preserves skeletal mechanical competence and calcium homeostasis; disturbance of this coupled turnover precipitated metabolic bone disease (Kim and Koh, 2019). Osteoporosis is defined by a reduction in bone mineral density (BMD) and increased skeletal fragility, thereby elevating fracture risk (Aslan, 2022). According to the World Health Organization guidelines, a dual-energy X-ray absorptiometry (DXA) *T*-score ≥−1.0 defines normal BMD; −1.0 to −2.5 indicates osteopenia; <−2.5 defines osteoporosis; and <−2.5 with a fragility fracture constitutes severe osteoporosis (Al-Hourani et al., 2021).

Bone turnover markers are categorized into two major groups. The initial category encompasses key bone formation markers, including serum alkaline phosphatase, osteocalcin, bone-specific alkaline phosphatase, procollagen type I N-terminal propeptide, procollagen type I C-terminal propeptide, and bone sialoprotein. Urinary hydroxyproline, urinary and serum type I collagen cross-linked N-telopeptide, serum type I collagen cross-linked C-telopeptide, and tartrate-resistant acid phosphatase (TRAP) fall into the second category of bone resorption markers, among others. These fracture-related biomarkers are valuable for diagnosing postmenopausal osteoporosis and evaluating therapeutic efficacy (Kanis et al., 2019; Vasikaran et al., 2011).

Data from a 2018 survey by China’s National Health Commission showed that the prevalence of osteopenia among women aged 40–49 years was 31.4%, increasing to 67.6% in women aged ≥50 years. In Japan, approximately 60% of postmenopausal women are classified as high-risk for osteoporosis (Iki, 2012). Similarly, a Korean study reported that osteoporosis prevalence among postmenopausal women reached 35–40%, approaching 50% in women aged ≥60 years (Lee et al., 2013). In India, prevalence rates vary widely, ranging from 8 to 62% (Agrawal and Garg, 2023). By 2050, it is projected that over 50% of global osteoporotic fractures will occur in Asia, emphasizing the urgent need for region-specific prevention strategies (Vijayakumar et al., 2025).

Currently, non-pharmacological interventions for maintaining BMD primarily involve calcium and vitamin D supplementation. However, accumulating evidence suggests that the gut microbiota is implicated in the regulation of bone metabolism through the gut-bone link (Hernandez et al., 2016; Bedree et al., 2023). Therefore, investigating the relationships between postmenopausal bone loss (*T*-score <−1.0), gut microbiota, serum biochemical markers, and serum metabolites offers remarkable potential for developing multi-pathway prevention and treatment strategies (Song et al., 2022). Nevertheless, the specific characteristics of gut microbiota and serum metabolites in postmenopausal females with osteopenia, and their correlations with bone quality, is poorly understood.

This study aimed to establish a scientific foundation for developing targeted prevention and treatment strategies for postmenopausal bone loss. Compared with prior studies that typically examined either gut microbiota or circulating metabolites alone, our study integrates 16S rRNA profiling and untargeted serum metabolomics in the same cohort and links multi-omic features to site-specific bone outcomes (L1–L4 *T*-scores). This integrative approach highlights a coherent association pattern involving NAA and the taxa Prevotella/Dorea, providing new clues to microbiota-metabolite-bone co-variation in postmenopausal bone loss.

## 2 Materials and methods

### 2.1 Study objectives

Participants were recruited from the Beijing region, China, with the following inclusion criteria: (1) postmenopausal women aged 40–75 years; (2) postmenopausal duration of at least 1 year; and (3) stable dietary habits and willingness to comply with study procedures.

Conversely, the exclusion criteria included: (1) individuals diagnosed with mental disorders, participants unable to accurately respond, unwilling to participate in the questionnaire survey, or unable to perform daily activities independently due to impaired recall, articulation, or movement disorders. (2) Individuals with degenerative or chronic bone diseases (e.g., osteomalacia, renal osteodystrophy, metabolic bone diseases, bone tumors) and traumatic fractures. (3) Individuals with systemic conditions with known skeletal implications, exemplified by diabetes mellitus, kidney stones, malignant tumors, hyperthyroidism, hypothyroidism, hyperparathyroidism, liver or kidney dysfunction, moderate-to-severe kidney dysfunction, characterized by serum creatinine concentrations >2 mg/dL or 177 μmol/L, abnormal liver function (alanine transaminase >2 × the upper limit of normal), chronic obstructive pulmonary disorder of moderate-to-severe grade, severe hypertension, or a previous diagnosis of cerebrovascular accidents. (4) History of digestive system surgery (e.g., gastrectomy, gastroplasty, and colostomy), persistent vomiting, or suspected gastrointestinal obstruction. (5) Administration of hormone therapy for the management of menopausal symptoms, anti-osteoporosis drugs, or bone metabolism-modulating agents (e.g., phenytoin, carbamazepine) within the past year, or treatment for symptomatic cholecystitis, active gastrointestinal ulcers, and a spectrum of urinary tract infections including pyelonephritis and cystitis, or thyroid dysfunction (e.g., hyperthyroidism) within the past 3 months. (6) Acute or chronic inflammatory diseases or current use of antibiotics, probiotics, prebiotics, or other medications capable of altering gut microbiota at enrollment; and (7) diagnosis of infectious diseases such as tuberculosis, acquired immunodeficiency syndrome, or severe organic diseases (e.g., malignant tumors, coronary heart disease, myocardial infarction, or stroke).

This study was conducted in accordance with the ethical standards set forth by the Ethics Committee of Beijing Ditan Hospital, Capital Medical University (Approval No.: Jing Di Lian Ke Zi [2022] (031)-02; approval date: July 1, 2022). All eligible participants provided written informed consent and received standardized training on questionnaire completion and fecal sample collection procedures.

### 2.2 Group allocation

*T*-scores of lumbar vertebrae (L1–L4) were measured using DXA for group allocation. Participants with a *T*-score <−1.0 were classified as the bone loss group (*n* = 58), whereas individuals with a *T*-score ≥−1.0 were assigned to the normal bone mass group (*n* = 47).

### 2.3 Collection and testing of baseline and clinical indicators

#### 2.3.1 Collection of baseline information

Participants filled out a baseline questionnaire containing demographic characteristics (age, height, weight, ethnicity) and clinical information (menopausal duration).

#### 2.3.2 BMD measurement

The BMD values of the lumbar vertebrae (L1–L4) and bilateral hip joints (i.e., femoral necks) were measured using a DXA scanner (DPX-L, Lunar Corp., Madison, WI, United States).

#### 2.3.3 Blood collection and laboratory testing

All participants underwent routine blood testing. Serum samples were collected for biochemical analysis and metabolomic profiling using vacuum blood collection tubes (three per participant). The tubes were then gently inverted 4-5 times to ensure proper mixing, incubated at 4 °C for 15–30 min prior to an 8 min centrifugation at 1200×g . The supernatant (pale yellow serum) was transferred to 5 mL sterile centrifuge tubes, and proteinase inhibitors were added at a ratio of 1:50 (inhibitor:serum) before thorough mixing. Serum aliquots were preserved at −80 °C until needed.

Serum bone metabolism markers were quantified using ELISA kits for parathyroid hormone (PTH; Nanjing Jiancheng Biotechnology Institute, Nanjing, China); insulin-mimetic growth factor-I, osteoprotegerin, along with receptor-activating agent of nuclear factor-κB ligand (RANKL; R&D Systems, MN, United States); and type I procollagen N-terminal pro-peptide, cross-linked C-telopeptide of type I collagen, osteocalcin, and TRAP isoform 5b (Guangzhou Feikang Biotechnology Co., Ltd., Guangzhou, China). A Beckman Coulter AU5800 automatic biochemical analyzer (Beckman Coulter, Inc., Brea, CA, United States) was utilized to assay the levels of serum alkaline phosphatase, calcium, and phosphorus. Serum 25-hydroxyvitamin D concentrations were determined using an AB SCIEX 6500 triple quadrupole liquid chromatography-coupled mass spectrometry (MS/MS) detection system (AB SCIEX, Framingham, MA, United States).

#### 2.3.4 Collection of fecal samples and sequencing of the 16S rRNA gene

Fecal specimens were gathered using sterile tubes (two per participant) to avoid urine contamination. Participants defecated into a sterile bedpan, and approximately 0.5–1.0 g of feces (from the middle and inner portions, 3–5 scoops per tube) was collected into the tube preservation solution. Within 48 h of collection, samples were kept at −20 °C before final transfer to a −80 °C freezer for archival purposes.

Fecal microbial DNA was recovered using the DP336 DNA extraction kit (Tiangen Biotech Co., Ltd., Beijing, China). Following quality verification (A260/A280 ratio 1.8–2.0), amplification of the 16S rRNA gene V3–V4 hypervariable regions was performed with specific primers. The PCR reaction system included Phusion^®^ High-Accuracy PCR Master Mix, forward and reverse oligonucleotide primers, and a genomic DNA template. The PCR cycling parameters were set as follows: 95 °C for 30 s (denaturation), 55 °C for 30 s (annealing), and 72 °C for 45 s (extension), with a total of 30 cycles performed. PCR products were purified with magnetic beads, pooled in equal amounts, and quality-checked. Qualified products were used for library construction and quantitative analysis. Library quality was confirmed by Qubit fluorometry and qPCR. An Illumina NovaSeq 6,000 platform (Illumina, Inc., San Diego, CA, United States) was utilized for sequencing with paired-end 250 bp (PE250) read length.

#### 2.3.5 Bioinformatic processing of 16S rRNA gene sequencing data

Raw paired-end sequencing reads were demultiplexed according to unique barcode and primer sequences. After removal of barcode and primer sequences, paired-end reads were merged using FLASH (version 1.2.11). The merged sequences were further trimmed using Cutadapt to remove residual primer sequences and adapters. Quality control was performed using fastp (version 0.23.1) to filter out low-quality reads and ambiguous bases, generating high-quality clean tags.

Chimeric sequences were identified and removed by comparison against the SILVA reference database using the UCHIME algorithm, resulting in effective tags for downstream analysis. Amplicon sequence variant (ASV) inference and denoising were conducted using the DADA2 algorithm implemented in QIIME2 (version 2022.02), which corrects sequencing errors and generates exact sequence variants without clustering.

Taxonomic classification was performed in QIIME2 using the SILVA database (release 138.1) for 16S rRNA gene sequences. SILVA is a well-curated and widely validated reference database for microbial community analysis. For taxa with incomplete hierarchical annotation in SILVA, taxonomic lineages were supplemented using NCBI taxonomy files. All samples were rarefied to the same sequencing depth prior to downstream diversity and differential abundance analyses.

#### 2.3.6 Serum metabolomic analysis

Metabolomic profiling of serum was performed using ultra-high-performance liquid chromatography-MS/MS. Briefly, 100 μL serum was mixed with 400 μL of 80% methanol (v/v) to precipitate proteins. By blending equal portions of all experimental samples, quality control samples were made. For blank samples, 53% methanol (v/v) was used instead of serum, followed by identical pretreatment conditions.

A Hypersil Gold C18 column (dimensions 2.1 × 100 mm, 1.9 μm, Thermo Fisher Scientific, Waltham, MA, United States) was used for chromatographic separation, with the column temperature set to 40 °C and a mobile phase flow rate of 0.2 mL/min. Using the positive ionic mode, the mobile phase comprised 0.1% (v/v) formic acid in water (phase A) and methanol (phase B). For the negative ion mode, the components included 5 mM ammonium acetate buffer (pH 9.0, phase A) and methanol (phase B). To boost separation efficacy, a gradient elution procedure was optimized.

The MS scanning was performed over *m*/*z* 100–1,500, and source parameters (spray voltage, sheath gas flow velocity, auxiliary gas flow velocity, along with ion transfer tube temperature) underwent optimization to attain peak ionization efficiency. Data-dependent acquisition mode was used for MS/MS data collection. Raw experimental data were introduced into Compound Discoverer 3.3 (CD 3.3) software (Thermo Fisher Scientific). Ion peaks were filtered by retention time and *m*/*z* values, and ion information was extracted and integrated following quality control sample calibration and parameter setting (e.g., mass deviation). Molecular formulas were deduced using parent and fragment ions, then matched to the Human Metabolome Database and MetLin. Background ions were subtracted using blank samples. Following peak area normalization, metabolites with a coefficient of variation >30% in quality control samples were excluded to ensure data reliability.

### 2.4 Statistical analysis

Statistical analyses were performed using Jamovi (version 2.3.28; The Jamovi project, Sydney, Australia) and R software (version 4.3.1; R Core Team, R Foundation for Statistical Computing, Vienna, Austria). Independent samples *t*-tests were applied to compare intergroup differences in normally distributed continuous variables.

For gut microbiota analysis, the vegan R package (Oksanen et al., 2025) was used to calculate α-diversity and β-diversity indices, with visualizations generated using the ggplot2 package (Hadley Wickham, Posit PBC, Boston, MA, United States). Intergroup differences in α-diversity were compared using independent samples *t*-tests, and β-diversity differences were assessed using the ADONIS2 function (permutation = 999). Differentially abundant gut microbial genera were identified using the Wilcoxon rank-sum test, and the resulting raw *p*-values were adjusted for multiple comparisons using the Benjamini–Hochberg (BH) false discovery rate procedure; corresponding plots created using GraphPad Prism (version 9.5; GraphPad Software, LLC, San Diego, CA, United States).

Linear discriminant analysis effect size (LEfSe) was conducted using the microeco R package (Chi Zhang, School of Ecological and Environmental Sciences, East China Normal University, Shanghai, China) with a linear discriminant analysis threshold of 1.2. Multivariate and metabolic pathway enrichment analyses of serum metabolites were performed using MetaboAnalyst (https://www.metaboanalyst.ca/; *Xia Lab*, McGill University, Montreal, Canada). Differentially abundant serum metabolites were identified based on a fold change >1.2 or <0.83 combined with *p* < 0.05, and visualized using ggplot2 (*Hadley Wickham*, Posit PBC).

Spearman’s rank correlation analysis was applied to examine the relationships between differentially abundant microbial genera, serum metabolites, and BMD *T*-scores. Only correlations with *p* < 0.05 and |*r*| ≥ 0.2 were retained. Correlation heatmaps were constructed using Origin 2024 (OriginLab Corporation, Northampton, MA, United States) and BioRender.com (Toronto, ON, Canada).

## 3 Results

### 3.1 Participants’ baseline information

A cohort of 105 volunteers from Beijing were enrolled in the study, including 47 participants in the normal bone density cohort and 58 within the bone loss group. The duration of menopause was considerably longer within the bone loss group than in the normal bone mass group (*p* = 0.017). No statistically marked variations were detected in baseline features—including age, height, and weight—between the two experimental groups (all *p* > 0.05; Table 1).

**Table 1:** Basic information.

Basic information	Normal group	Bone loss group	*p*-value
Age (years)	60.17 ± 10.59	61.96 ± 18.48	0.075
Height (cm)	160.25 ± 5.48	158.83 ± 7.97	0.131
Weight (kg)	65.50 ± 12.11	61.77 ± 11.22	0.066
Duration of menopause (years)	9.76 ± 10.93	12.56 ± 11.93	0.017*
Ovariectomy (cases)	Not ovariectomized/ovariectomized: 43/4	Not ovariectomized/ovariectomized: 54/4	0.908

### 3.2 Bone status

Bone mass-related parameters were collected from all participants. Compared with the normal bone mass, the bone loss group exhibited significantly lower *T*-scores for the left total femur (*p* = 0.041), right femoral neck (*p* = 0.039), and right entire femur (*p* = 0.04). Similarly, BMD values for lumbar vertebrae L1–L4 (*p* = 0.001), left total femur (*p* = 0.048), right femoral neck (*p* = 0.038), and right total femur (*p* = 0.038) were significantly lower in the bone loss group. The *Z*-score corresponding to lumbar vertebrae L1–L4 was also considerably lower within the bone loss group (*p* = 0.001; Table 2).

**Table 2:** Bone quality information.

Bone quality information	Normal group	Bone loss group	*p*-value
Left femoral neck *T*-score	−1.03 ± 0.22	−1.41 ± 0.27	0.064
Total left femur *T*-score	−0.69 ± 0.22	−1.08 ± 0.24	0.041*
Right femoral neck *T*-score	−1.05 ± 0.24	−1.39 ± 0.18	0.039*
Total right femur *T*-score	−0.70 ± 0.22	−1.04 ± 0.17	0.04*
L1–L4 BMD value	1.06 ± 0.14	0.87 ± 0.14	0.001**
Left femoral neck BMD value	0.81 ± 0.02	0.76 ± 0.04	0.433
Total left femur BMD value	0.89 ± 0.02	0.83 ± 0.04	0.048*
Right femoral neck BMD value	0.80 ± 0.02	0.76 ± 0.02	0.038*
Total right femur BMD value	0.88 ± 0.20	0.84 ± 0.02	0.036*
L1–L4 *Z*-value	0.84 ± 0.25	−0.48 ± 0.2	0.001**
Left femoral neck *Z*-value	−0.030 ± 0.24	−0.21 ± 0.25	0.433
Total left femur *Z*-value	−0.49 ± 1.53	−0.24 ± 1.75	0.46
Right femoral neck *Z*-value	−0.07 ± 0.26	−0.20 ± 0.18	0.515
Total right femur *Z*-value	−0.032 ± 0.24	−0.21 ± 0.18	0.301

*p < 0.05; **p < 0.01; ***p < 0.001.

### 3.3 Metabolite characteristics

#### 3.3.1 Serum biochemical markers

In total, 104 volunteers (46 with normal bone mass and 58 in bone loss group) were included in the serum biochemical marker analysis. Among the bone loss group, the mean serum levels of 25-hydroxyvitamin D (25(OH)D; *p* = 0.001) and 25-hydroxyvitamin D3 (25(OH)D3; *p* = 0.001) were markedly higher than those in the normal bone mass group (Table 3).

**Table 3:** Blood biochemical information.

Blood biochemical indicators	Normal group	Bone loss group	*p*-value
CR (μmol/L)	55.30 ± 2.63	53.91 ± 2.55	0.46
ALP (U/L)	71.46 ± 5.38	67.88 ± 4.77	0.33
ALT (U/L)	21.87 ± 3.63	22.05 ± 3.36	0.47
AST (U/L)	22.35 ± 2.42	20.96 ± 1.85	0.26
Calcium (Ca) (mmol/L)	2.35 ± 0.05	2.36 ± 0.05	0.25
Phosphorus (P) (mmol/L)	1.05 ± 0.04	1.07 ± 0.03	0.16
25(OH)D3 (ng/mL)	13.14 ± 1.34	15.59 ± 1.36	0.01**
25(OH)D2 (ng/mL)	1.20 ± 0.37	1.42 ± 0.74	0.92
25(OH)D (ng/mL)	14.34 ± 1.34	17.01 ± 1.54	0.01**
TRACP5b (U/L)	1.79 ± 0.16	1.90 ± 0.16	0.25
CTX (ng/ml)	0.33 ± 0.07	0.39 ± 0.08	0.39
PINP (ng/ml)	83.69 ± 10.50	90.00 ± 12.60	0.99
PTH (ng/L)	86.13 ± 6.27	87.49 ± 6.91	0.83
OC (ng/mL)	24.27 ± 2.55	22.37 ± 2.08	0.29
RANKL (pg/mL)	13.52 ± 7.54	29.70 ± 28.14	0.83
IGF-1 (ng/mL)	10.77 ± 1.74	9.71 ± 1.45	0.44

#### 3.3.2 Serum metabolite characteristics

**Figure 1:**
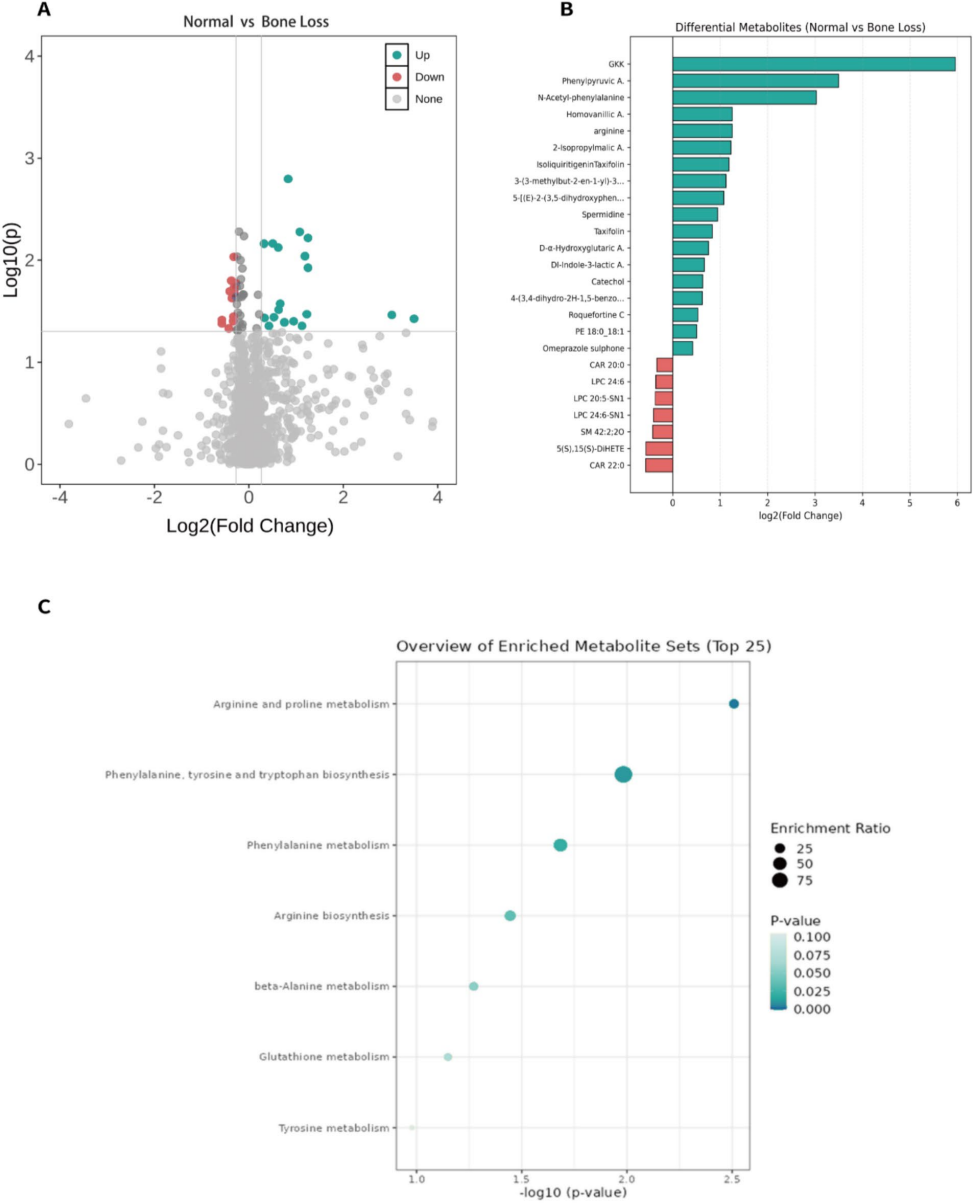
Serum metabolomics distinguishes postmenopausal bone loss and highlights NAA as a key correlate. **(A)** Volcano plot of differential serum metabolites between the normal bone mass group and the bone loss group (FC = normal/bone loss). Metabolites meeting the predefined criteria (FC > 1.2 or <0.83 and *p* < 0.05) are highlighted. Key metabolites (e.g., NAA, GKK, and taxifolin) are annotated. **(B)** Fold-change distribution (log_2_FC) of significantly differential metabolites, ranked by absolute effect size. **(C)** KEGG pathway enrichment of differential metabolites (MetaboAnalyst). Pathways with nominal *p* < 0.05 are shown; none remained significant after FDR correction (all *q* > 0.1), therefore results are considered exploratory.

Serum metabolomic profiling was conducted for 104 participants (46 with normal bone mass and 58 with bone loss). Among the 1,347 detected metabolites, 33 were differentially abundant (Figure 1A). Compared with the bone loss group, the normal bone mass group exhibited 20 upregulated and 13 downregulated differential metabolites (Supplementary Table 1). In addition, GKK (Gly-Lys-Lys), a small peptide detected in the untargeted metabolomic analysis, showed one of the strongest contributions to group discrimination based on fold change analysis (Figure 1B), although its biological role remains largely unclear.

Specifically, flavonoids such as taxifolin and isoliquiritigenin, as well as phenolic compounds including the hydroxystilbene derivative 5-[(E)-2-(3,5-dihydroxyphenyl)ethenyl]-2-methoxybenzene-1,3-diol and homovanillic acid, were significantly upregulated (*p* < 0.05). The phospholipid phosphatidylethanolamine 18:0_18:1 level was also significantly upregulated (*p* < 0.05), whereas the levels of the phosphatidylcholine O-18:3, lysophosphatidylcholine (LPC) 20:5-SN1, and lysophosphatidylcholine (Lysopc) 20:4 were significantly downregulated (*p* < 0.05). In addition, amino acids and their metabolites—including L-arginine, spermidine (an arginine-derived polyamine), phenylpyruvic acid, and N-acetyl-L-phenylalanine—were significantly upregulated (*p* < 0.05) as was the organic acid NAA (*p* < 0.05). Kyoto Encyclopedia of Genes and Genomes metabolic pathway enrichment analysis (Figure 1C) identified several pathways associated with these metabolites, including arginine and proline metabolism [*p* = 0.003, enrichment ratio = 21.3%, false discovery rate (FDR) *q* = 0.248], phenylalanine metabolism (*p* = 0.025, enrichment ratio = 48.0%, FDR *q* = 0.551), phenylalanine, tyrosine, and tryptophan biosynthesis (*p* = 0.012, enrichment ratio = 96.15%, FDR *q* = 0.415), and arginine biosynthesis (*p* = 0.036, enrichment ratio = 27.47%, FDR *q* = 0.719). Although these pathways met the unadjusted significance threshold (*p* < 0.05) and demonstrated high enrichment ratios, all FDR-adjusted *q*-values exceeded 0.1.

### 3.4 Gut microbiota analysis

**Figure 2:**
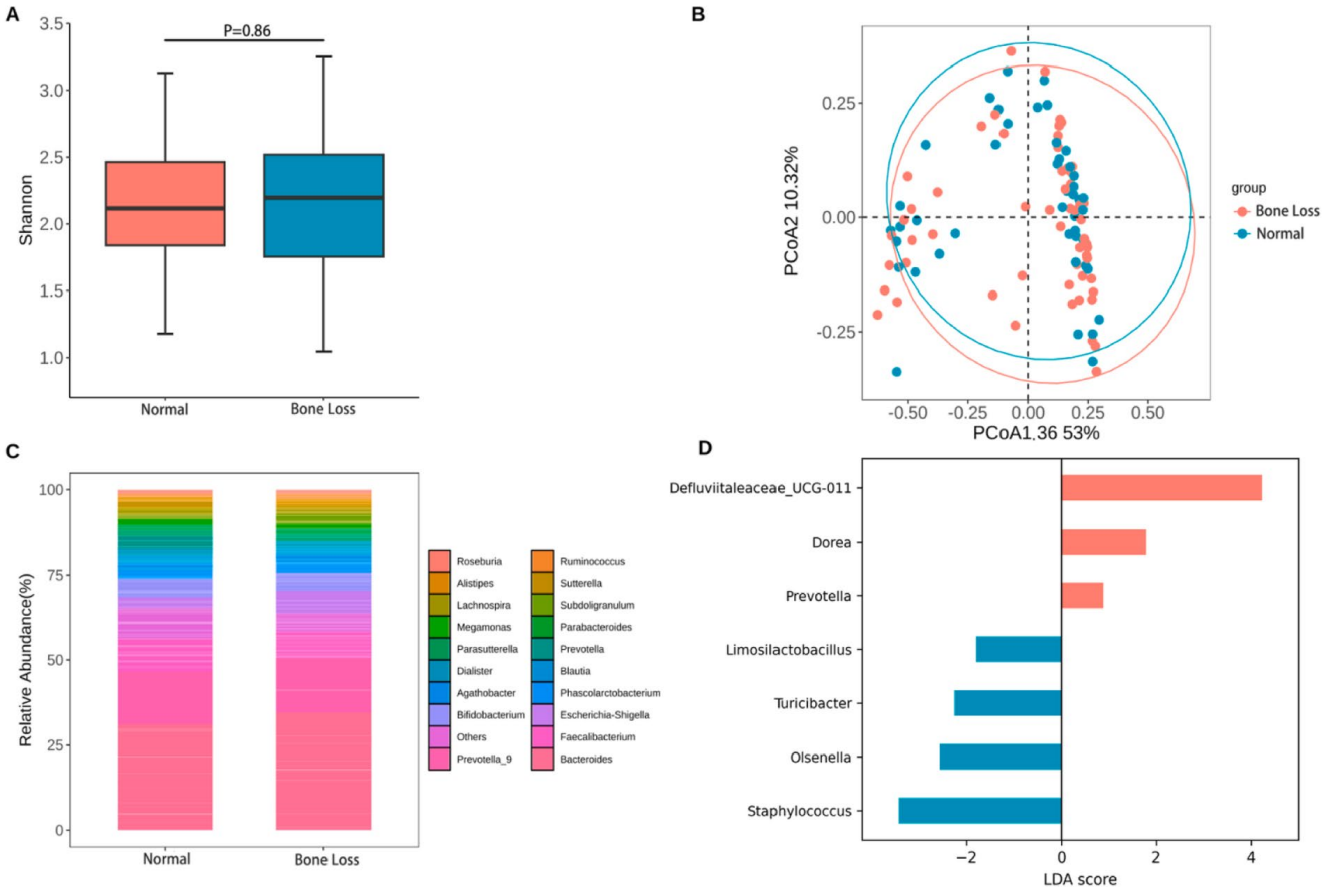
Global gut community structure shows no marked separation between groups, while LEfSe identifies group-enriched taxa. **(A)** Alpha diversity (Shannon Index) of fecal microbiota in the normal bone mass and bone loss groups (*n* indicated in the plot). **(B)** Beta diversity assessed by PCoA based on Bray–Curtis dissimilarity, with group separation tested by PERMANOVA (ADONIS2, 999 permutations). **(C)** Relative abundance of the top 20 genera; “Others” includes remaining low-abundance taxa. **(D)** LEfSe analysis (LDA threshold = 1.2) showing taxa differentially enriched between groups.

Fecal samples were collected from 103 participants (46 in the normal bone mass group and 57 in the bone loss group). No statistically notable intergroup variations were found in *α*-diversity (Shannon Index; Figure 2A *p* = 0.86), β-diversity (principal coordinates analysis; Figure 2B), or dominant microbiota abundance (Figure 2C; *p* > 0.05) (Supplementary Tables 2,3).

**Figure 3:**
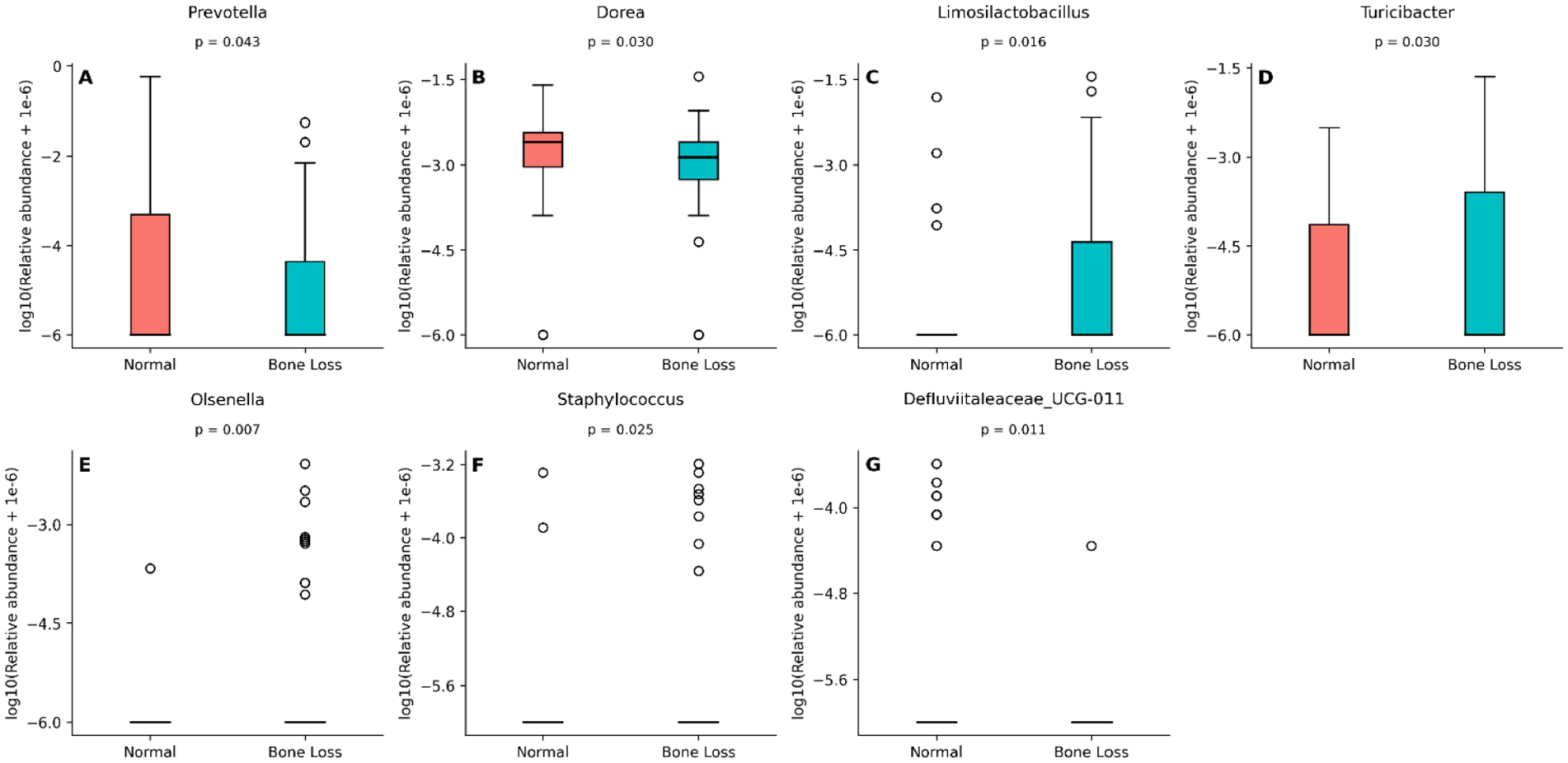
Seven genera differ between the normal bone mass and bone loss groups. Box-and-jitter plots show the relative abundances of genera identified by LEfSe and confirmed by Wilcoxon rank-sum tests. **(A)** *Prevotella*, **(B)** *Dorea*, **(C)** *Limosilactobacillus*, **(D)** *Turicibacter*, **(E)** *Olsenella*, **(F)** *Staphylococcus*, and **(G)** *Defluviitaleaceae_UCG-011*. Genera enriched in the normal bone mass group include *Prevotella*, *Dorea*, and *Defluviitaleaceae_UCG-011*, whereas *Limosilactobacillus*, *Olsenella*, *Turicibacter*, and *Staphylococcus* are enriched in the bone loss group. For visualization, genus-level relative abundances were log_10_-transformed as log_10_ (relative abundance + 1×10⁻⁶). *P* values are indicated in each panel.

LEfSe (Figure 2D; LDA score >1.2) was used for feature selection, and the Wilcoxon rank-sum test (Figure 3) was used to confirm group differences and report *p*-values, identifying seven genera with intergroup differences (Supplementary Table 4). Relative to the normal group, the bone loss group exhibited considerably lower relative abundance of *Prevotella* (*p* = 0.042), *Dorea* (*p* = 0.030), and *Defluviitaleaceae_UCG-011* (*p* = 0.01), with *Prevotella* showing the most pronounced difference. By contrast, the bone loss group exhibited significantly higher relative representation of *Limosilactobacillus* (*p* = 0.015), *Olsenella* (*p* = 0.007), *Turicibacter* (*p* = 0.029), and *Staphylococcus* (*p* = 0.024). However, after BH correction for multiple comparisons, none of these genera remained statistically significant (all *q* > 0.05); indicating that these signals should be interpreted as exploratory.

### 3.5 Correlation analysis

Rank correlation analysis using Spearman’s method was conducted to examine associations between differential serum metabolites, blood biochemical markers, and gut microbiota indices across all participants (Normal and Bone Loss groups), using the L1–L4 *T*-score as the bone status indicator.

The L1–L4 *T*-score showed significant negative correlations with 25(OH)D3 (Spearman’s *r* = −0.23, *p* = 0.016) and with 25(OH)D (*r* = −0.24, *p* = 0.011). Defluviitaleaceae_UCG-011 was positively correlated with the *T*-score corresponding to L1–L4 lumbar vertebrae (*r* = 0.287, *p* = 0.0034), whereas Limosilactobacillus (*r* = −0.27, *p* = 0.005), Olsenella (*r* = −0.22, *p* = 0.025), and Staphylococcus (*r* = −0.20, *p* = 0.046) were negatively correlated. Dorea (*r* = 0.188, *p* = 0.058) and Prevotella (*r* = 0.183, *p* = 0.065) showed positive but non-significant correlations with the L1–L4 *T*-score. In addition, the relative abundances of Prevotella and Dorea were not significantly correlated (*r* = 0.015, *p* = 0.880).

**Figure 4:**
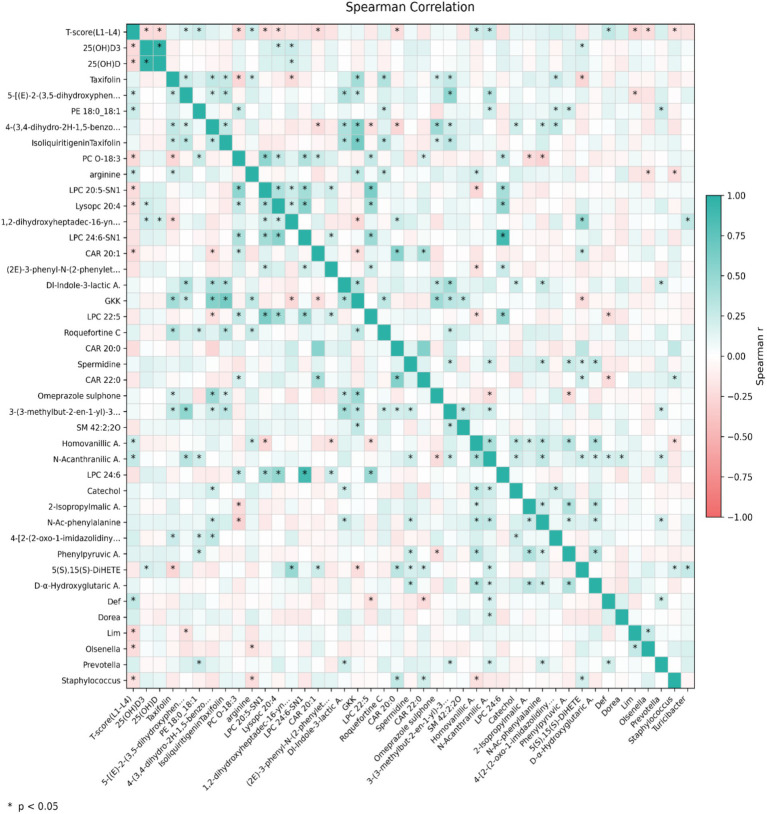
Integrated correlation map links differential gut taxa, serum metabolites, and lumbar bone status. Spearman correlations were computed among differential genera, differential serum metabolites, selected biochemical indicators, and the L1–L4 *T*-score (Supplementary Table 5). Only correlations with *p* < 0.05 and |*r*| ≥ 0.2 were retained and visualized. Color indicates the correlation coefficient (*r*). Key associations include the positive correlation of NAA with L1–L4 *T*-score and with *Prevotella*/*Dorea*.

Additionally, the L1–L4 *T*-score correlated with several differential serum metabolites (Figure 4). Specifically, NAA demonstrated a positive correlation relative to the L1–L4 *T*-score (r = 0.27, *p* < 0.005) and with *Defluviitaleaceae_UCG-011* (*r* = 0.20, *p* = 0.043), *Dorea* (*r* = 0.21, *p* = 0.028), and *Prevotella* (*r* = 0.20, *p* = 0.042). It also linked to the differential metabolite catechol (*r* = 0.22, *p* = 0.021), N-acetyl-L-phenylalanine (*r* = 0.28, *p* = 0.0034), and D-*α*-hydroxyglutaric acid (*r* = 0.20, *p* = 0.043).

## 4 Discussion

The key new insight from this work is the integrative multi-omics correlation map linking gut taxa and serum metabolites with lumbar bone status. In particular, NAA showed a positive association with L1–L4 *T*-scores and co-varied with Prevotella and Dorea, suggesting a new potential microbiota–metabolite model of bone metabolic regulation that warrants further mechanistic validation.

Decreased estrogen levels in postmenopausal women disrupted bone remodeling, leading to an imbalance in which bone resorptive processes exceed osteogenic activity and induces persistent bone loss. In this study, the “normal group” and “bone loss group” represent different stages of this process. The lower *T*-scores observed in the bone loss group reflected the cumulative effect of long-term remodeling imbalance, indicating that bone mass has entered the osteopenia or osteoporosis range. Some researchers have argued that osteoporosis should not be strictly defined as a disease since rigid diagnostic thresholds may prevent treatment for individuals who do not meet the criteria but are at high fracture risk. Instead, bone loss can be regarded as a common process affecting most individuals from around 50–60 years of age onward (Reid and Billington, 2022). Therefore, the osteopenia and osteoporosis groups were unified as a single bone loss group, reflecting a continuous and progressive pathological process. By comparing differences in gut microbiota, bone turnover biomarkers, blood biochemical indices, and serum metabolomic profiles between the osteopenia and normal groups—and analyzing their correlations with bone mass—this study identified potential diagnostic, preventive, and therapeutic strategies for postmenopausal bone loss.

Compared with the normal group, the bone loss group exhibited a markedly longer menopause duration, independent of age. Estrogen deficiency following prolonged menopause accelerates bone breakdown and inhibits osteogenesis, ultimately culminating in bone depletion (Melis et al., 2025). Bone status assessments revealed that the *T*-scores and BMD values for the total left femur, neck of the right femur, and overall right femoral region proved significantly reduced among the bone loss group than in the normal bone mass group, indicating that bone loss substantially reduces BMD. A significant difference in *Z*-score was only found at the L1–L4 vertebrae; all other sites showed no significant variations. This may be because the *Z*-score reflects bone density relative to individuals of the same age, sex, and ethnicity; no considerable discrepancies were noted due to the sample in this study being highly homogeneous (Muhan et al., 2022).

Blood biochemical analysis revealed that 25(OH)D and 25(OH)D3 concentrations were higher in the bone loss group than in the normal group; however, absolute levels in both groups remained below commonly used reference ranges for sufficiency. Correlation analysis further showed significant inverse associations between circulating 25(OH)D/25(OH)D3 and the L1–L4 *T*-score (25(OH)D3: *r* = −0.23, *p* = 0.016; 25(OH)D: *r* = −0.24, *p* = 0.011). These cross-sectional findings may reflect altered vitamin D metabolism, supplementation or health-seeking behaviors, and/or reduced receptor sensitivity (e.g., lower VDR expression), rather than a direct pathogenic effect of vitamin D (Anderson et al., 2017). In principle, activation of the PTH axis can increase renal 1α-hydroxylase activity and promote the conversion of 25(OH)D to 1,25(OH)2D3, thereby enhancing intestinal calcium absorption; however, in our cohort PTH levels were similar between groups. Importantly, because the study is cross-sectional and vitamin D concentrations were subnormal overall, causal interpretation is not warranted. Longitudinal investigations are required to clarify the relationships among vitamin D metabolism, receptor sensitivity, and bone loss rates.

Overall, the serum metabolomic profile of women with normal bone status differed from that of the bone-loss group, characterized by coordinated changes across multiple metabolite classes relevant to bone remodeling. Specifically, flavonoids such as taxifolin and isoliquiritigenin were relatively higher in the normal group, a pattern that is directionally consistent with prior experimental evidence linking these compounds to reduced osteoclastogenic signaling and bone-resorptive activity (Zhang et al., 2019; Liu et al., 2016). In addition, several amino acids and related metabolites—including L-arginine and the polyamine spermidine—as well as aromatic amino acid derivatives, were more abundant in the normal group; these observations are broadly consistent with reported links between amino acid metabolic axes and osteoblast–osteoclast balance, although such mechanistic interpretations remain indirect in an observational setting (Riancho et al., 1995; Lee et al., 2017; Izawa et al., 2016; Voronov et al., 2008). Phospholipid alterations were also evident: phosphatidylethanolamine (PE 18:0_18:1) was higher in the normal group, whereas multiple LPC species were lower. Given that membrane phospholipid composition may influence cellular functions relevant to mineralization and signaling, these findings may reflect a metabolic milieu more favorable to bone homeostasis (Fisk and Kano-Sueoka, 1992; Agrawal et al., 2011).

Among the differential metabolites, NAA emerged as a key correlate. NAA was higher in the normal group and positively associated with the L1–L4 *T*-score, while also co-varying with the relative abundances of Prevotella and Dorea. Notably, although both genera were associated with NAA, their relative abundances were not significantly correlated, suggesting that they might contribute to the metabolite pool through independent or complementary pathways rather than synchronized expansion. Prior evidence suggests that NAA may act as an endogenous ligand involved in aryl hydrocarbon receptor-related pathways that are relevant to osteoclast/osteoblast regulation (Park et al., 2020). Taken together, these results highlight a microbiota-metabolite signature linked to lumbar bone status in our cohort and provide a testable hypothesis for microbiota–metabolite–bone crosstalk. However, given the cross-sectional design and genus-level microbiota resolution, the directionality and mechanistic basis of these associations require confirmation in longitudinal cohorts and functional studies.

Pathway enrichment analysis suggested several nominally enriched pathways (e.g., arginine/proline metabolism and aromatic amino acid-related pathways), but these signals did not remain significant after FDR correction (all *q* > 0.1). Therefore, pathway-level interpretations should be regarded as exploratory and primarily hypothesis-generating, and the lack of FDR significance likely reflects limited statistical power in a moderate-sized cohort. Notably, GKK (Gly-Lys-Lys), a small peptide detected in the untargeted metabolomic analysis, showed one of the stronger contributions to group discrimination based on fold-change patterns; however, its biological relevance to bone metabolism remains unclear at present. Thus, GKK is more appropriately interpreted as a marker of altered peptide/amino acid metabolism in the bone-loss state, warranting further validation in larger cohorts and targeted functional studies.

The gut microbiota-bone link is known to regulate bone metabolism (Tu et al., 2023; Lyu et al., 2023). Our analysis revealed no significant disparities in α- and β-diversity across the groups, genus-level analysis revealed higher *Prevotella* abundance in the normal group. As *Prevotella* may protect against estrogen-deficiency–induced bone loss (Wang et al., 2021; Zhang et al., 2023) by influencing carbon, branched-chain amino acids, as well as bone metabolism through the citric acid cycle (Lu et al., 2024). Additionally, the normal group exhibited higher *Dorea* abundance than that of the bone loss group, whereas *Limosilactobacillus*, significantly enriched in the bone loss group, exhibited an inverse association with the L1–L4 *T*-score (*r* = −0.27). Although Prevotella (*r* = 0.183, *p* = 0.065) and Dorea (*r* = 0.188, *p* = 0.058) tended to correlate positively with the L1–L4 *T*-score, these associations were not statistically significant and therefore warrant cautious interpretation. Studies of spaceflight and simulated microgravity have reported increased Dorea and Limosilactobacillus abundance in rodent intestines, accompanied by shifts in circulating metabolites relevant to bone remodeling (e.g., short-chain fatty acids) and evidence of trabecular bone loss and increased bone marrow adipogenesis (Bedree et al., 2023). However, other studies found that certain *Lactobacillus* spp. have a bone-improving effect. *Lactobacillus reuteri* administration increased femoral and lumbar trabeculae bone volume fraction and BMD in male mice, and prevented bone loss in ovariectomized mice (Villa et al., 2017). This contradiction may stem from the functional heterogeneity of bacterial species/strains and environmental influences. Since this study analyzed only genus-level data, metagenomic sequencing is required to clarify the species-grade specificity.

We detected a significant positive correlation between Olsenella (enriched in the bone loss group) and Limosilactobacillus. Both genera showed negative correlations with the L1–L4 *T*-score, suggesting the possible symbiotic regulation of bone metabolism. The negative correlation between Staphylococcus and the L1–L4 *T*-score may reflect an opportunistic expansion of inflammation-associated taxa, potentially contributing to a pro-inflammatory milieu. *Staphylococcus* may indirectly influence bone metabolism through inflammation resulting from gut microbiota alterations (Waldbaum et al., 2023). Overall, because genus-level differences did not remain significant after BH correction (all *q* > 0.05), the taxa discussed above should be regarded as exploratory candidates.

**Figure 5:**
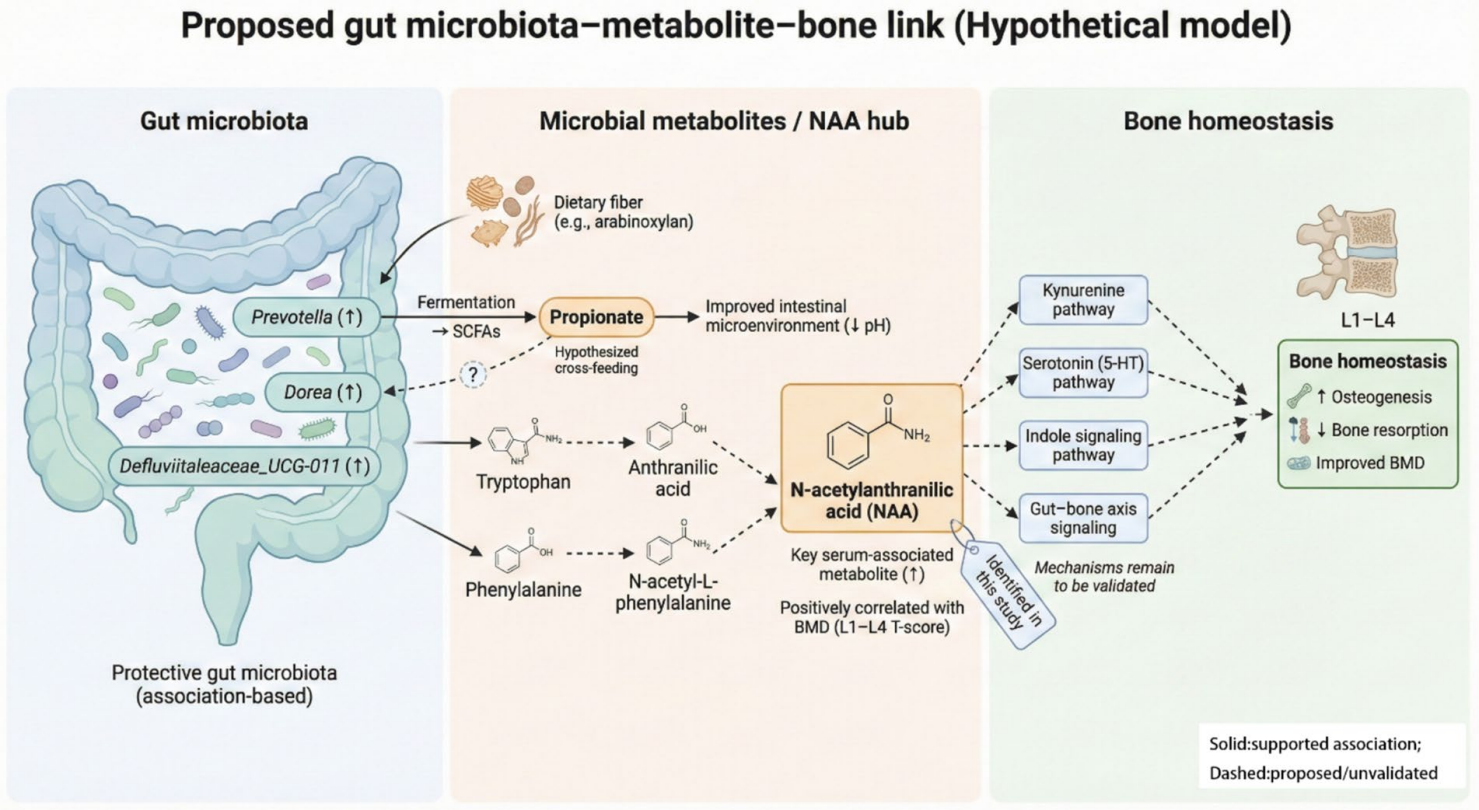
Proposed gut microbiota-metabolite-bone link (hypothetical model). This figure summarizes an association-based, hypothesis-generating model derived from a cross-sectional cohort of postmenopausal women grouped by DXA-derived lumbar status (L1–L4 *T*-score ≥−1.0, normal; <−1.0, bone loss). In this cohort, *Prevotella*, *Dorea*, and *Defluviitaleaceae_UCG-011* were reduced in the bone-loss group and co-varied with the serum metabolite NAA, which was positively correlated with L1–L4 *T*-scores. Fiber fermentation to SCFAs (propionate) is shown as an evidence-supported association, whereas cross-feeding and aromatic amino acid conversion steps are depicted as proposed pathways because genus-level 16S data cannot confirm functional capacity. Potential downstream host pathways (kynurenine, serotonin/5-HT, indole signaling, gut-bone signaling) are included as mechanistic hypotheses requiring validation. Solid arrows indicate evidence-supported associations observed in this study (differential abundance and/or correlation), whereas dashed arrows indicate proposed or literature-based mechanistic links that were not directly tested here and require further validation.

The differential serum metabolite NAA was significantly positively correlated with the L1–L4 *T*-score for BMD and protective genera *Prevotella*, *Dorea*, *Defluviitaleaceae_UCG-011*, and N-acetyl-L-phenylalanine (Figure 4). Therefore, gut microbiota may be involved in NAA production and function via a synergistic metabolic network, influencing bone homeostasis (Figure 5). Mechanistically, *Prevotella* degraded dietary fiber (e.g., arabinoxylan) to produce short-chain fatty acids such as propionate (Bartsch et al., 2025). Propionate provided energy for host colonic cells and may optimize the growth environment for *Dorea* by reducing intestinal pH (Brinck et al., 2025); however, in our cohort Prevotella and Dorea were not significantly correlated in relative abundance (*r* = 0.015, *p* = 0.880), This lack of direct correlation suggests that their contributions to the NAA-associated bone protection likely reflect functional complementarity within the gut-bone axis, rather than a synchronized expansion of these two genera. Certain fungi expressed anthranilate synthase, a precursor of NAA, which converts tryptophan to anthranilic acid (Zhao et al., 2025). Genes encoding enzymes involved in aromatic amino acid metabolism (including phenylalanine deamination) have been reported in several gut bacterial taxa, such as Enterococcus and Clostridium (Shen et al., 2022; Alili et al., 2022). However, it is also possible that Dorea harbors similar functional capacity. This possibility cannot be confirmed from genus-level 16S data alone and should be validated using shotgun metagenomics and/or isolate-based functional assays. Although the specific metabolic pathways of *Defluviitaleaceae_UCG-011* in the context of bone health are not yet fully elucidated, its consistent co-variation with bone phenotypes and protective metabolites suggests that it may be a key member of the microbial network contributing to the NAA metabolic hub. As an acetylated tryptophan derivative (Paul and Ratledge, 1971), NAA participates in tryptophan metabolism, thereby potentially modulating osteochondrocyte activity through multiple signaling pathways, including the receptor mediation, intercellular coupling, gut-bone link crosstalk (kynurenine), serotonin (5-HT), and indole pathways (Xiang et al., 2025). Cross-regulation between phenylalanine and tryptophan metabolism may represent a core mechanism linking NAA to bone homeostasis. The positive correlation between NAA and N-acetyl-L-phenylalanine suggests that phenylalanine and tryptophan metabolism may form a synergistic regulatory network by sharing intermediate products, such as anthranilic acid (Figure 5), by working together to maintain the dynamic equilibrium between osteogenesis and resorption.

### 4.1 Study limitations

This study has several limitations. First, the relatively small cohort size and all participants being from Beijing limited generalizability. Second, the metabolomics pathway-level associations did not remain significant after FDR correction (all *q* > 0.05), necessitating validation in larger cohorts and through longitudinal or experimental studies. Similarly, genus-level differential taxa identified at the nominal level did not survive BH correction (all *q* > 0.05) and should therefore be interpreted as exploratory, which may reflect modest effect sizes, substantial inter-individual heterogeneity, and the multiple-testing burden in a relatively small cohort, as well as the limited taxonomic/functional resolution of genus-level 16S profiling. Finally, due to the cross-sectional design, a causal relationship between the gut microbiota and bone loss cannot be established, highlighting the need for longitudinal and interventional studies. Additionally, unmeasured lifestyle factors (diet composition, physical activity, and sunlight exposure) may influence both the gut microbiota and circulating metabolome, and residual confounding cannot be excluded.

### 4.2 Future research directions

The mechanisms underlying these findings should be validated by verifying the direct regulatory effects of *Prevotella* and *Dorea* on NAA synthesis and bone density using germ-free mouse models. Additionally, metagenomic sequencing should be employed to determine the species composition of *Limosilactobacillus* and *Dorea*. Future clinical translation involved exploring the non-pharmacological interventions, including dietary fiber or probiotic interventions to modulate NAA levels and construct an integrated multi-omics “microbiota-metabolite-bone density” predictive model.

## 5 Conclusion

In this cross-sectional cohort, postmenopausal bone loss was associated with specific gut microbial and serum metabolic signatures. The co-variation of NAA with Prevotella/Dorea and L1–L4 *T*-scores provides a testable hypothesis and potential candidate biomarkers for future longitudinal and interventional studies.

## Data Availability

The original contributions presented in this study are included in this article/Supplementary material, further inquiries can be directed to the corresponding authors. Additionally, the microbiome data have been deposited in the NCBI BioProject database (https://www.ncbi.nlm.nih.gov/bioproject), under accession number PRJNA1401517.
