# Supplementary figures and images for: Altered gut microbiota and serum metabolite profiles characterize postmenopausal bone loss: insights into the gut-bone axis
Source: Front Microbiol. 2026 Mar 4;17:1750495. doi: 10.3389/fmicb.2026.1750495 (PMC12996171; doi:10.3389/fmicb.2026.1750495)

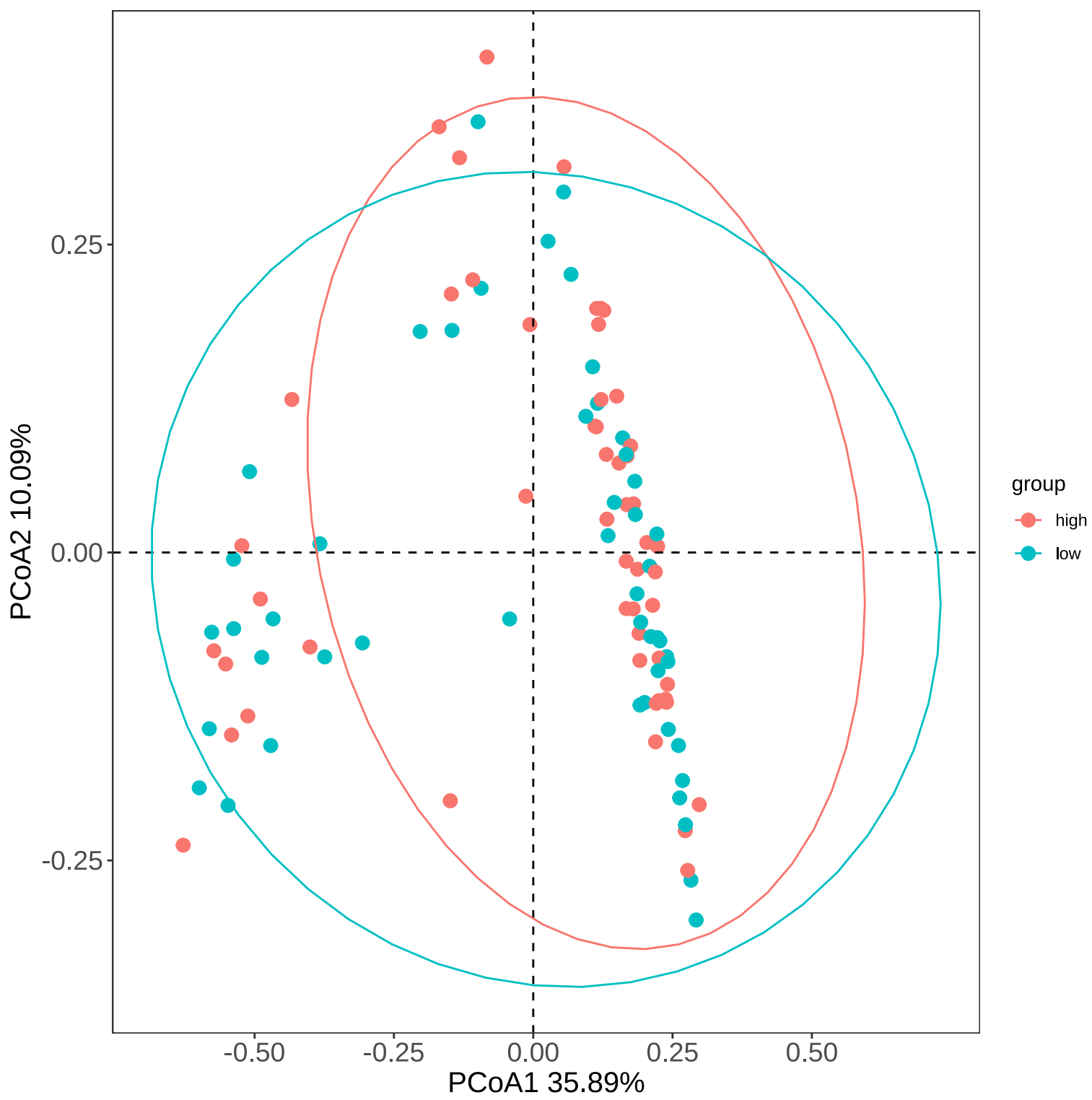

Supplement: Supplementary file 7 [file Data_Sheet_1.pdf]

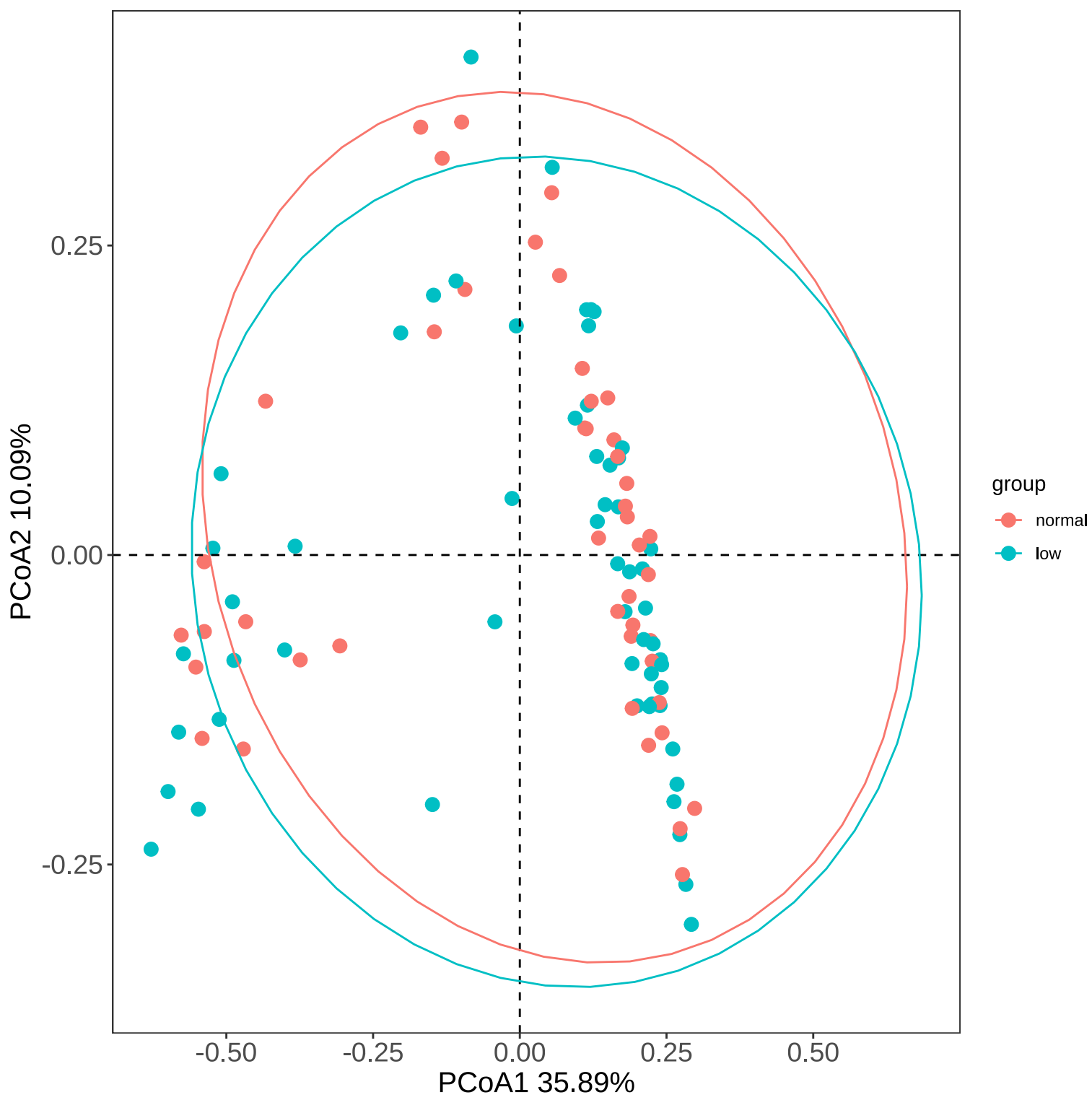

Supplement: Supplementary file 8 [file Data_Sheet_2.pdf]

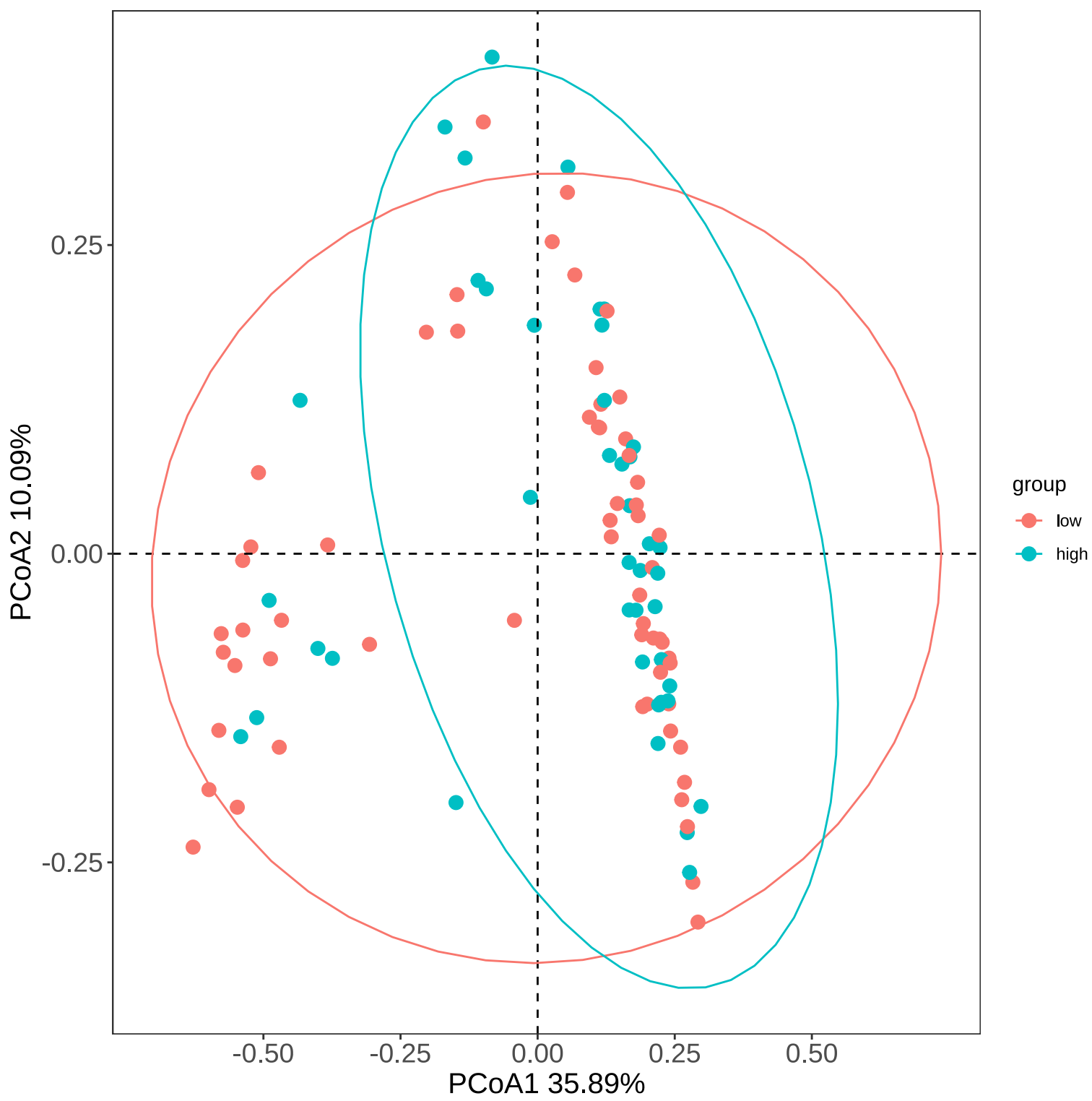

Supplement: Supplementary file 9 [file Data_Sheet_3.pdf]

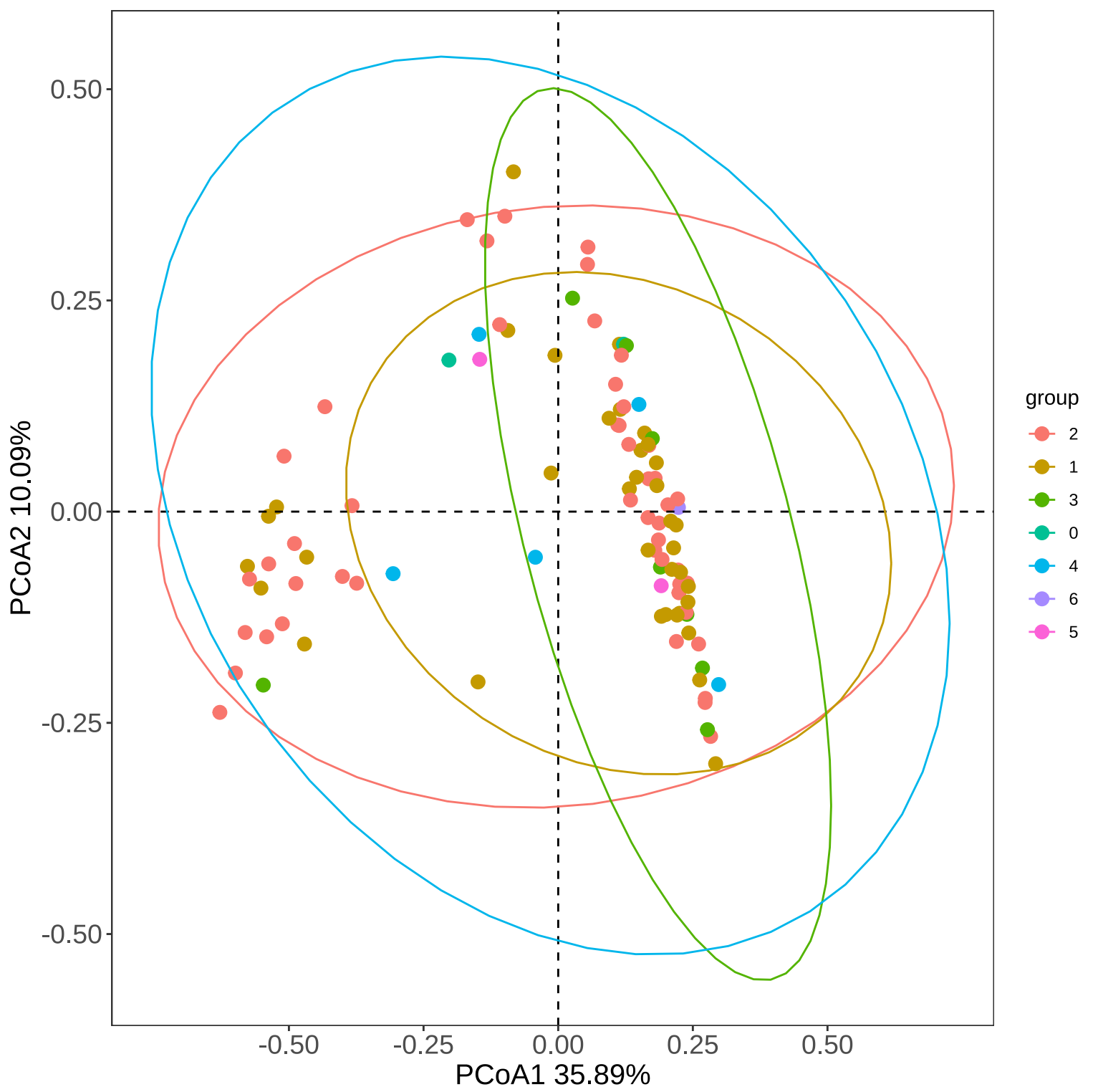

Supplement: Supplementary file 10 [file Data_Sheet_4.pdf]

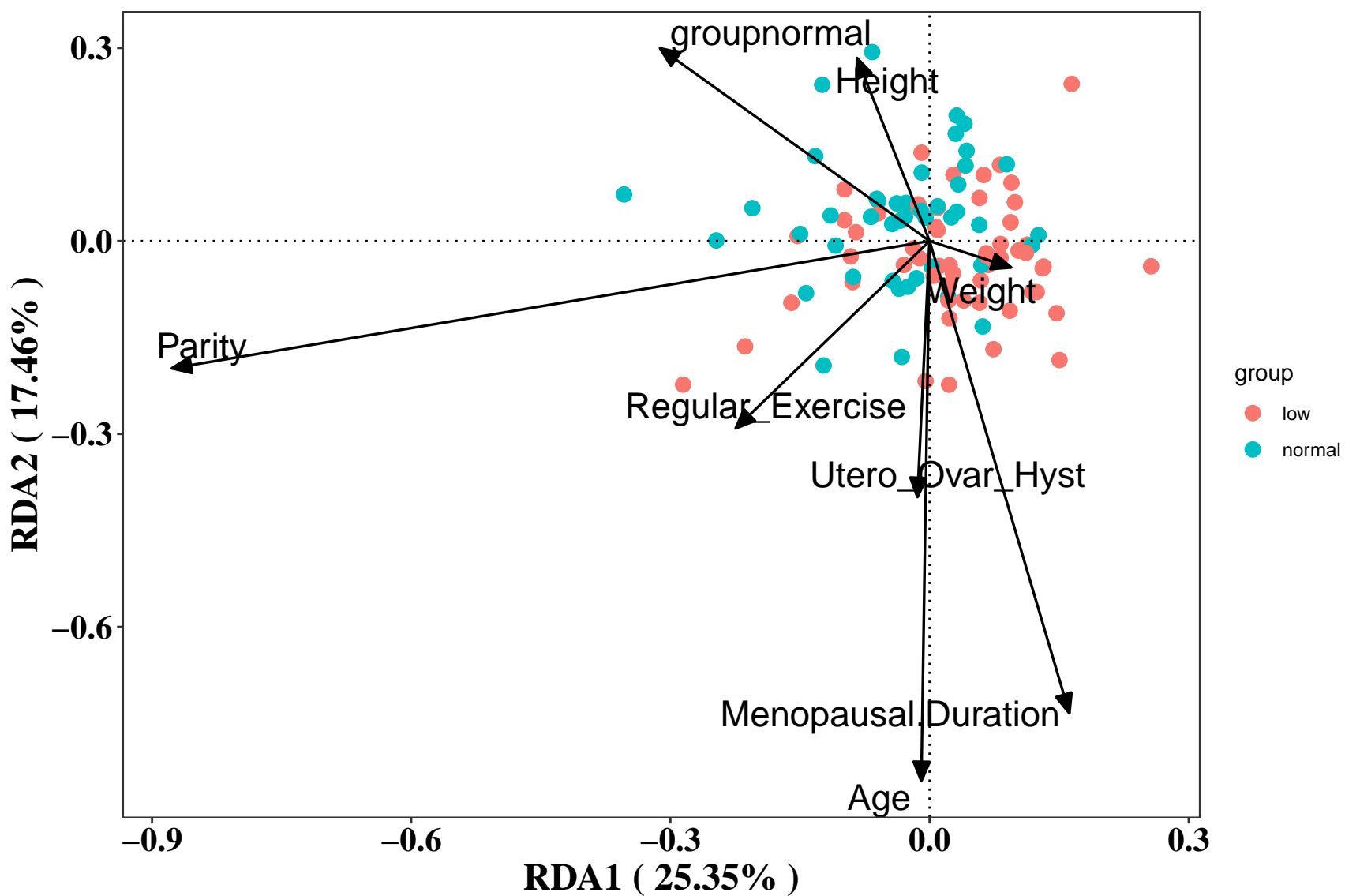

Supplement: Supplementary file 11 [file Data_Sheet_5.pdf]

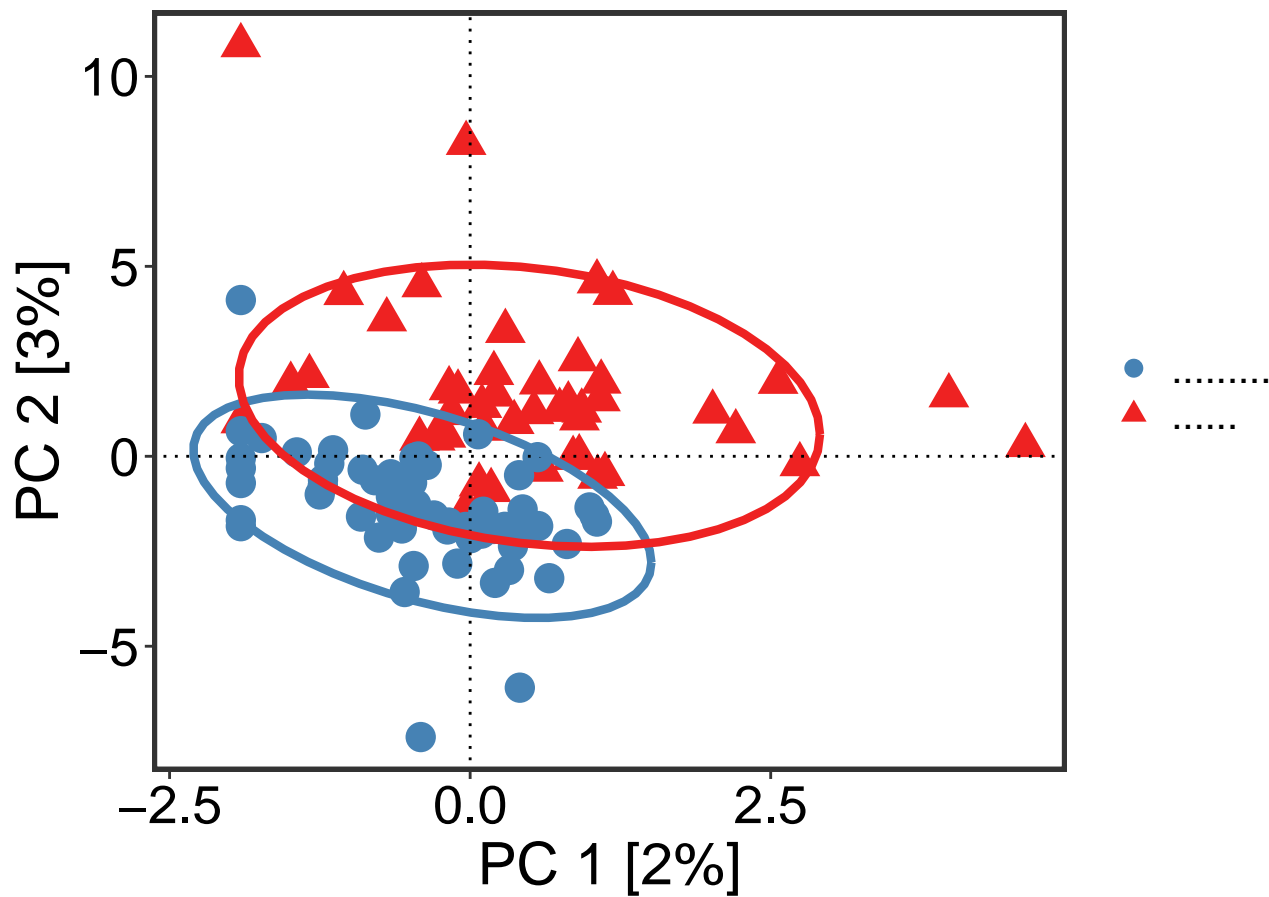

Supplement: Supplementary file 13 [file Data_Sheet_7.pdf]

■ Negative ■ Positive

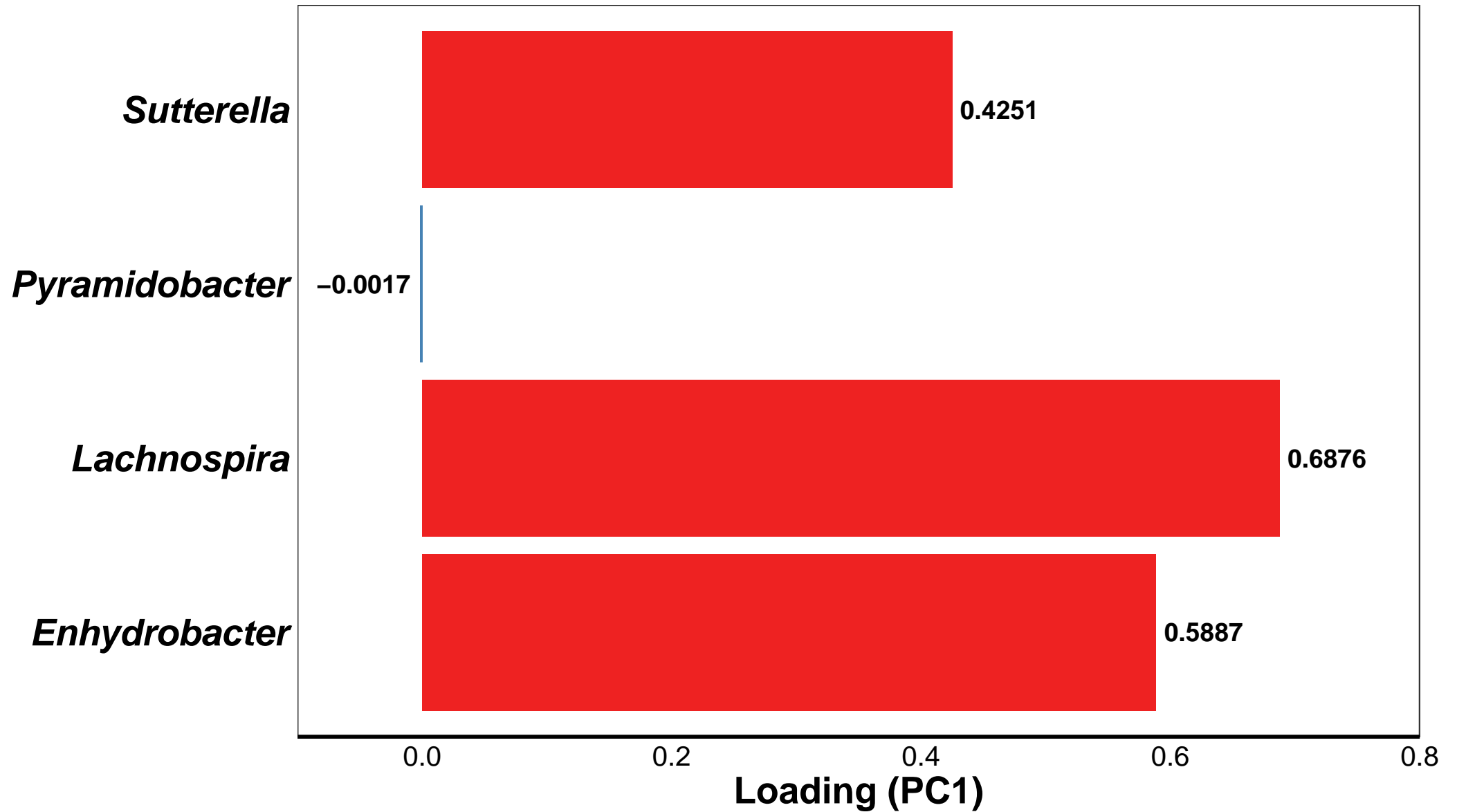

Supplement: Supplementary file 14 [file Data_Sheet_8.pdf]
