# Supplementary material for: Altered gut microbiota and serum metabolite profiles characterize postmenopausal bone loss: insights into the gut-bone axis
Source: Front Microbiol. 2026 Mar 4;17:1750495. doi: 10.3389/fmicb.2026.1750495 (PMC12996171; doi:10.3389/fmicb.2026.1750495)
Supplement: Supplementary file 12 [file Data_Sheet_6.pdf]

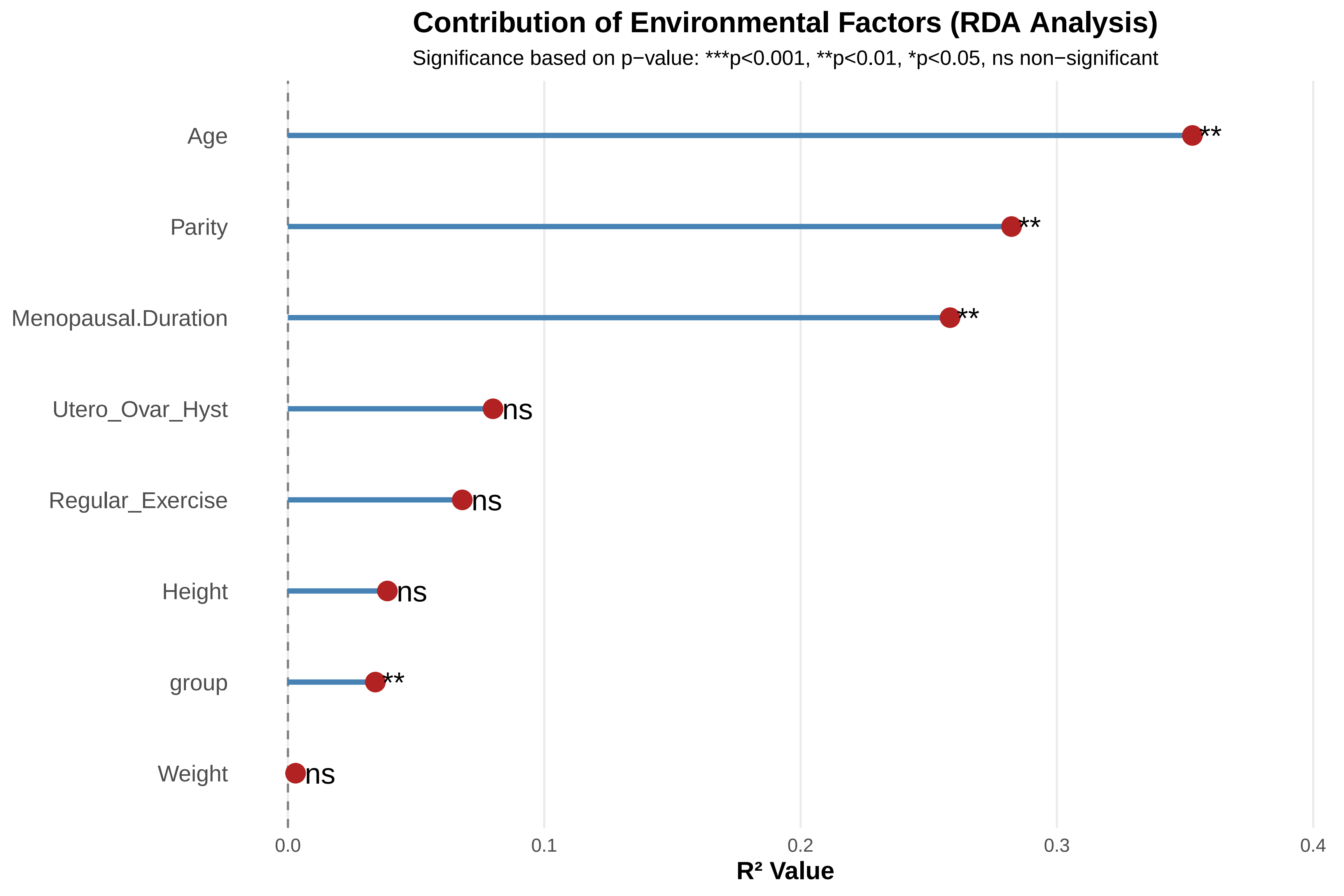

Contribution of Environmental Factors (RDA Analysis)
Significance based on p−value: ***p<0.001, **p<0.01, *p<0.05, ns non−significant
Age
Parity
Menopausal.Duration
Utero_Ovar_Hyst
Regular_Exercise
Height
group
Weight
**
**
**
ns
ns
ns
**
ns
0.0
0.1
0.2
0.3
0.4
R² Value
